# p53-independent early and late apoptosis is mediated by ceramide after exposure of tumor cells to photon or carbon ion irradiation

**DOI:** 10.1186/1471-2407-13-151

**Published:** 2013-03-25

**Authors:** Gersende Alphonse, Mira Maalouf, Priscillia Battiston-Montagne, Dominique Ardail, Michaël Beuve, Robert Rousson, Gisela Taucher-Scholz, Claudia Fournier, Claire Rodriguez-Lafrasse

**Affiliations:** 1 Université de Lyon, Lyon, F-69622, France; 2 Faculté de Médecine-Lyon-Sud, Université LyonI, Oullins, F-69921, France; 3 Laboratoire de Radiobiologie-Cellulaire-et-Moléculaire, EMR3738 Lyon1-HCL, Oullins, France; 4 Hospices-Civils-de-Lyon, Centre-Hospitalier-Lyon-Sud, Pierre-Bénite, France; 5 Hospices-Civils-de-Lyon, Groupe Hospitalier Est, Bron, France; 6 IPNL-LIRIS-CNRS-IN2P3, Villeurbanne, France; 7 Biophysics-Department, GSI Helmholtz Center for Heavy-Ion-Research, Darmstadt, Germany

**Keywords:** Ceramide, Carbon ion irradiation, High- and low-LET-irradiation, Early and late apoptosis, p53-independent-apoptosis

## Abstract

**Background:**

To determine whether ceramide is responsible for the induction of p53-independent early or late apoptosis in response to high- and low-Linear-Energy-Transfer (LET) irradiation.

**Methods:**

Four cell lines displaying different radiosensitivities and p53-protein status were irradiated with photons or 33.4 or 184 keV/μm carbon ions. The kinetics of ceramide production was quantified by fluorescent microscopy or High-Performance-Liquid-Chromatogaphy and the sequence of events leading to apoptosis by flow cytometry.

**Results:**

Regardless of the p53-status, both low and high-LET irradiation induced an early ceramide production in radiosensitive cells and late in the radioresistant. This production strongly correlated with the level of early apoptosis in radiosensitive cells and delayed apoptosis in the radioresistant ones, regardless of radiation quality, tumor type, radiosensitivity, or p53-status. Inhibition of caspase activity or ceramide production showed that, for both types of radiation, ceramide is essential for the initiation of early apoptosis in radiosensitive cells and late apoptosis following mitotic catastrophe in radioresistant cells.

**Conclusions:**

Ceramide is a determining factor in the onset of early and late apoptosis after low and high-LET irradiation and is the mediator of the p53-independent-apoptotic pathway. We propose that ceramide is the molecular bridge between mitotic catastrophe and the commitment phase of delayed apoptosis in response to irradiation.

## Background

To date a large majority of radiobiological studies have claimed that the primary and major relevant target of irradiation is the nucleus and particularly the DNA. However selective irradiation of the cytoplasm [1] induces cell radiosensitivity, proving that an onset of cell signaling originates from other organelles than the nucleus. Moreover, ionizing-radiation can act directly on free-nucleus membrane preparations, generating ceramide. These data suggest that the membrane represents an alternative target to DNA in radiation-induced-cell-response [2]. Although ionizing-radiation and ceramide can separately promote death, cell cycle arrest or differentiation, ceramide-induced signaling after irradiation has been mostly linked to apoptosis. Moreover a lack of ceramide production has been reported to be correlated with radioresistance [3,4]. Indeed, radioresistance of Head-and-Neck-Squamous-Carcinoma-Cell (HNSCC) cells was associated with a lack of *acid-*Sphingomyelinase translocation and activation to the plasma membrane thus a lack of ceramide generation leading to the absence of formation of signaling platforms [5] and then the absence of apoptotic signaling. Ceramide, as p53, is then emerging as a fundamental mediator of apoptosis. However relationships existing between these two molecules are still controversial and need to be clarified. Several reports suggest that ceramide accumulation is an important downstream mediator of the p53 response whereas others have shown that p53 and ceramide are concomitantly up-regulated in response to various cell-stressors and that ceramide can accumulate and signal for apoptosis, irrespective of p53 status [6]. Moreover the relationship between ceramide and the different types of apoptosis needs to be defined. Apoptosis can occur before the first mitosis (early apoptosis) or as the last step of mitotic catastrophe [7] (late apoptosis). Although the involvement of ceramide in the response to photon exposure is well characterized in early apoptosis, its involvement in late apoptosis, or last step of mitotic catastrophe as well as its role in the triggering of cell death after exposure to different radiation qualities such as carbon ion irradiation needs to be strongly clarified.

Hadrontherapy or carbon ion therapy is a new emerging and promising therapy which can offer several advantages over conventional radiotherapy because of the physical and biological properties of the particles. Taking advantage of these clinically relevant properties, a high number of patients with glioblastoma or HNSCC tumor have been treated with carbon ions, and the results were found to be very promising [8]. To date the exact mechanisms leading to apoptotic cell death in response to high-LET-radiation are only partially known. High-LET-radiation is more effective than low-LET-radiation in inducing apoptosis in cancer cells from different origins and displaying different p53-status [9-11]. Nevertheless the exact mechanism or pathways involved after high-LET exposure have never been described up to now. The few available data only suggest that high-LET irradiation can induce apoptosis through a p53-independent pathway involving the activation of caspase-9, which subsequently leads to the cleavage of caspase-3 [12,13]. Only in one study, α-particles were used to show an induction of apoptosis through the sphingomyelin pathway [14].

In a previous study [4] we demonstrated that, in response to photon irradiation, ceramide induces early-apoptosis through caspase activation, independently of p53, in radiosensitive cells. By contrast when ceramide is not generated, the whole pathway is ineffective inducing a resistance to apoptosis. Moreover, we reported, in the same radiosensitive cells, that carbon irradiation induces early apoptosis. By contrast, the radioresistant cells underwent mitotic catastrophe followed by late apoptosis 5 days after irradiation [11]. Given these incomplete results and regarding the role of the ceramide apoptotic pathway, the aim of this study was to determine the pivotal role of ceramide in early and late apoptosis after exposure of cells with different radiosensitivities, p53-status and tumor of origin to different types of irradiation.

## Methods

### Cell culture

Two radiosensitive cell lines, SCC61 [11] and SF767 [15], and two radioresistant cell lines, SQ20B [11] and U87MG [15], were used (Table 1). HNSCC SCC61 and SQ20B cells, were grown as previously described [11]. Glioblastoma U87MG and SF767 cells were grown in DMEM, 10% Fetal-Calf-Serum, 100U/ml penicillin, 100 mg/ml streptomycin, 1 mM sodium pyruvate, and 0.1 mM nonessential amino-acids.

**Table 1:** Radiobiological parameters and p53 status for SCC61, SQ20B, SF767 and U87MG cell lines

	**Tumor types**	**P53 status**	**SF2**			**D10**		
			**Photon**	**Carbon 33.4 keV/μm**	**Carbon 184 keV/μm**	**Photon**	**Carbon 33.4 keV/μm**	**Carbon 184 keV/μm**
**SCC61**	HNSCC	−/−	0.36	0.14	0.036	3.05	2.3	1.35
**SQ20B**	HNSCC	−/−	0.72	0.21	0.13	6.2	3	2.1
**SF767**	Glioma	+/+	0.45	0.16	0.083	4.7	2.4	1.85
**U87MG**	Glioma	+/+	0.81	0.24	0.15	6.5	3.2	2.1

### Irradiation procedure and pharmacological treatment

Sixteen hours before irradiation, cells were seeded on 25 cm^2^ flasks or 6-well plates at a concentration varying between 3.10^4^ to 2.10^6^ depending on the kinetics and on the dose of irradiation as previously described [11].

Irradiation using 6MV photons, 75 MeV/u carbon ions (LET:33.4 keV/μm), and 11.4 MeV/u carbon ions (LET:184 keV/μm) was performed at Lyon-Sud Hospital, Grand-Accélérateur-National-d’Ion-Lourds (France) and Helmholtzzentrum-für-Schwerionenforschung (Germany) as described previously [11].

The total caspase inhibitor Z-VAD-fmk (50 μM) was added to the medium 2 h before and 48 and 120 h after irradiation. Ceramide production was inhibited either by 1 mM chloroalanine, 10 μM Fumonisin B1, 50 μM imipramine or by 20 μM desipramine, which was added to cells 30 min before irradiation and 48 and 120 h following irradiation.

### Flow cytometry analysis

The percentage of cells in each phase of the cell cycle was quantified after propidium iodide labeling as previously described [11].

The terminal-transferase-dUTP-nick-end-labeling (TUNEL) reaction was carried out by labeling cells with the *in situ* Cell-Death-Detection-Kit (Promega, France) according to the manufacturer’s instructions.

For the measurement of transmembrane mitochondrial potential, the cells were trypsinized and incubated with 5 μg/ml JC-1 (5,5’,6,6’-tetrachloro-1,1’,3,3’tetraethylbenzimidazolylarbo-cyanine-iodide) for 20 min at 37°C. The cells were rinsed, resuspended in PBS, and assessed for red and green fluorescence using flow cytometry. A minimum of 10,000 cells were analyzed.

Caspase activation was quantified using the CaspACE™ FITC-VAD-FMK In Situ Marker kit (Promega, France) according to the manufacturer’s instructions.

### Ceramide quantification

Early ceramide quantification was realized using fluorescent microscopy as described in [16]. Images were captured by a Zeiss Axio Imager Z2 using Metafer software. Fluoresence intensity, reported to the cell size, was quantified using Metafer software (Metasystems, Germany). A minimum of 600 cells were scored on 2 independent slides and the mean was calculated. Late ceramide production was quantified by HPLC with fluorimetric detection, as described previously [4].

### Statistical analysis

Student’s *t* test was used to compare values between groups.

## Results

### Involvement of ceramide production in the response to low- and high-LET radiation

The kinetics of intracellular ceramide production was assessed after exposure of the four HNSCC or glioblastoma cells, displaying different radiosensitivity and p53-status [11,15] (Table 1), to 10Gy irradiation with photons (low LET) or with 2 high LET carbon ions (33.4 or 184 keV/μm). The same physical dose of 10Gy and non-biological-equivalent-dose was used since we sought to investigate potentially different mechanisms which may become clearer and should better emphasize the differences between the two types of beams applied.

**Figure 1:**
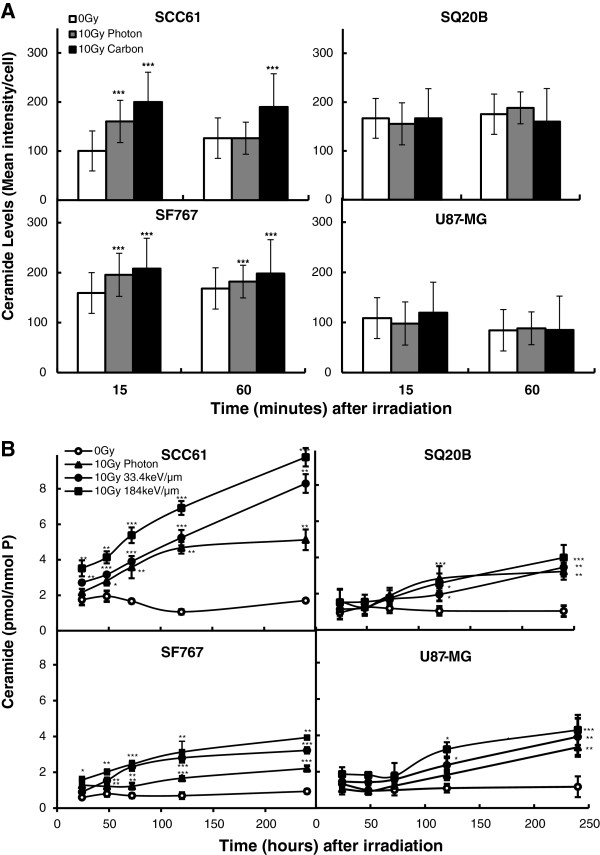
**Time course of ceramide production in SCC61, SF767, SQ20B, and U87MG cell lines after a 10Gy irradiation with photons or 33.4 or 184 keV/μm carbon ions.** Early ceramide production (**A**) was quantified by fluorencence microscopy and late ceramide (**B**) by HPLC. Values represent the mean ± SD of two or three independent experiments performed in triplicate. *p < 0.05, **p < 0.01, and ***p < 0.001 compared to control.

According to the kinetics, two different methods for the ceramide quantification were used. For the early ceramide production (less than 1 hour) an immunofluorescent staining of permeabilized cells using a ceramide-specific antibody was used [16] whereas HPLC detection was applied for the late ceramide production. Results obtained for the early ceramide quantification are shown in Figure 1A. Two different pattern of response were observed. For both radiosensitive cell lines (SCC61 p53 mutated and SF767 p53 wild type), a significant ceramide production occurred with a very early peak at 15 min after both high and low LET irradiation. As an example, for SCC61 the mean intensity of fluorescence goes from 100 to 160 after photon irradiation. This ceramide release is more pronounced after carbon ion irradiation (mean intensity = 200) and is prolonged until 1 hour post irradiation. At the opposite, no ceramide increase was observed for the radioresistant U87MG and SQ20B cell lines whatever the type of irradiation used. Concerning the late ceramide production, in the radiosensitive SCC61 p53-mutated cells, irradiation induced a time-and LET-dependent increase in ceramide level compared with non-irradiated cells (Figure 1B). Ceramide concentration increased from 24 h up to 240 h. At 240 h the amount of ceramide obtained was 5.1, 8.3, and 9.8 pmol/nmolP following photon, 33.4 keV/μm or 184 keV/μm carbon irradiation, respectively. The same pattern of response was observed in the p53-wild-type radiosensitive SF767 cells. Irradiation with 33.4 keV/μm carbon ions increased ceramide concentration that started at 24 h and reached 4.7 pmol/nmolP at 240 h, whereas the value was 5.6 pmol/nmol P after 184 keV/μm. In contrast to this, ceramide concentration in the p53-mutated SQ20B resistant cells began to increase significantly only 120 h after irradiation. At 240 h, ceramide amount was 2.9, 3.2, and 3.9 pmol/nmol P following photon, 33.4 keV/μm or 184 keV/μm carbon irradiation, respectively. In the p53-wild-type radioresistant U87MG cell line, the kinetics and ceramide concentration were similar to those measured in SQ20B cells.

### Activation of apoptosis through mitochondrial dysfunction and caspase activation by low-and high-LET radiation

**Figure 2:**
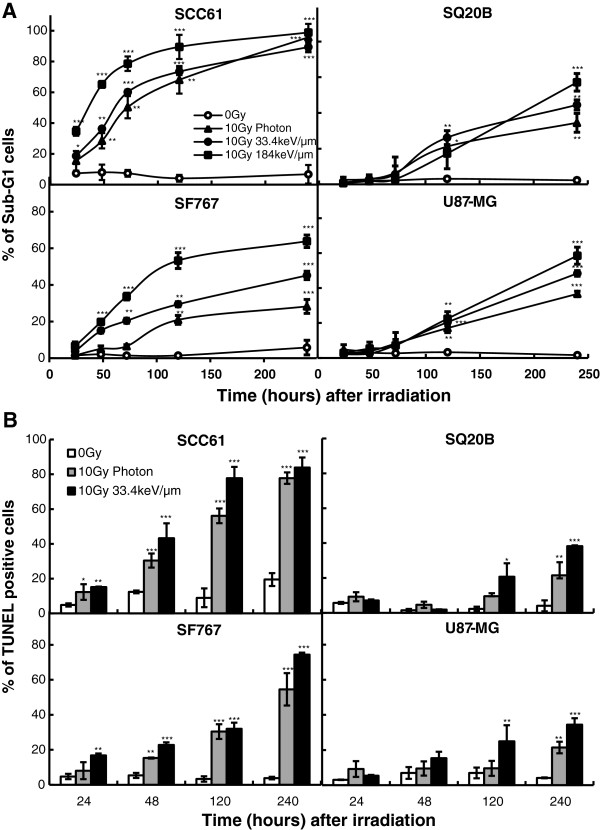
**Kinetics of apoptosis. A**: quantified as the percentage of cells in the sub-G1 phase, in the four cell lines irradiated with 10Gy photons or with 33.4 or 184 keV/μm carbon ions. **B**: Kinetics of TUNEL positive cells, in the four cell lines irradiated with 10Gy photons or with 33.4 carbon ions. Values represent the mean ± SD of two or three independent experiments performed in triplicate *p < 0.05, **p < 0.01, and ***p < 0.001 compared to control.

In order to determine if low-and high-LET-radiation can induce apoptosis, four different techniques were used to monitor apoptosis: the quantification of sub-G1 and TUNEL positive cells, the mitochondrial dysfunction and the activation of caspases. Early apoptosis was quantified in radiosensitive cells, starting from 24 h after irradiation and increasing significantly with both time and LET (Figure 2A). In SCC61 cells, the percentages of sub-G1 were 50.3 ± 7.2% at 72 h after photon irradiation and, 60.1 ± 1.3% after 33.4 keV/μm and 78.4 ± 8.7% after 184 keV/μm carbon irradiation. In SF767 cells, apoptosis increased slightly later and was 20.4 ± 1.4% and 33.4 ± 1.9% at 72 h following 33.4 and 184 keV/μm carbon irradiation respectively. In SQ20B and U87MG radioresistant cells, no significant induction of apoptosis was observed during the first 72 h following low-or high-LET irradiation. Late apoptosis was only significant from 120 h and reached levels ranging between 30% and 50%, depending on radiation quality. Regardless of the type of radiation, apoptotic levels were always lower in radioresistant compared with radiosensitive cells. In order to confirm these results, TUNEL analyses were realized with the four cell lines, from 24 to 240 h after photons or 33.4 keV/μm carbon irradiation (Figure 2B). As expected, this experiment confirmed the results obtained after the analysis of the sub-G1 peak. For both SCC61 and SF767 cells, an early apoptosis was observed whereas a late apoptosis appears for SQ20B and U87 cell lines.

**Figure 3:**
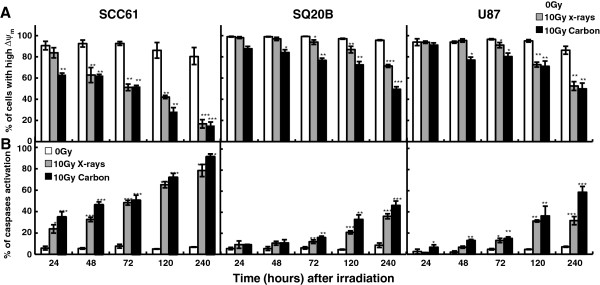
**Kinetics of mitochondrial membrane potential (A) and total caspase activation (B) in SCC61, SQ20B, and U87MG cell lines after exposure to photons or to 184 keV/μm carbon irradiations.** Values represent the mean ± SD of two independent experiments performed in triplicate *p < 0.05, **p < 0.01, and ***p < 0.001. compared to control.

In order to ascertain the potential involvement of the intrinsic apoptotic pathway, a kinetic study of the mitochondrial transmembrane potential (ΔΨm) and caspase activation were both analyzed by flow cytometry. As depicted in Figure 3A, exposure of SCC61 cells to radiation induced an early LET-and time-dependent decrease in ΔΨm. At 24 h, the percentage of cells displaying a high ΔΨm were 83.9 ± 4.8% after photon exposure and 63.3 ± 1.4% after 184 keV/μm carbon exposure, whereas ΔΨm decreased to about 15% at 240 h. In SQ20B and U87MG cells, the decrease in ΔΨm began from 120 h and felt to about 50% of the initial value at 240 h. As depicted in Figure 3B a time-and LET-dependent activation of total caspases was observed which started 24 h after irradiation in SCC61 cells whatever the type of radiation applied. In radioresistant cells, the activation of caspases was much more limited, delayed in time since it began to significantly increase only 120 h after irradiation.

In order to confirm that late apoptosis was activated in radioresistant cells as the last step of mitotic catastrophe, the percentage of cells arrested in the G2/M phase and the percentage of polyploid cells were quantified by flow cytometry.

**Figure 4:**
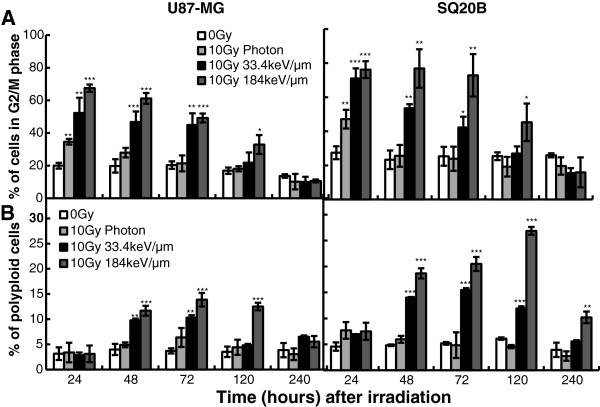
**Mitotic catastrophe induction in U87MG and SQ20B cells. Upper panel**: Percentage of U87MG and SQ20B cells in G2/M phase as a function of time after irradiation **Lower panel**: Kinetics of the occurrence of polyploid SQ20B cells after irradiation with X-rays or carbon beams. Values represent the mean ± SD of three independent experiments performed in triplicate *p < 0.05, **p < 0.01, and ***p < 0.001 compared to control.

Figure 4 clearly shows that in response to carbon or photon exposure, both radioresistant cells undergo a G2/M phase arrest starting from 24 h up to 120 h (Upper panel). Moreover a marked increase of polyploid cells, a characteristic event of mitotic catastrophe, was demonstrated after both types of irradiation (Lower panel). These results strongly suggest that delayed apoptosis is effectively the last event of mitotic catastrophe.

### Functional relationship between ceramide production and induction of apoptosis

**Figure 5:**
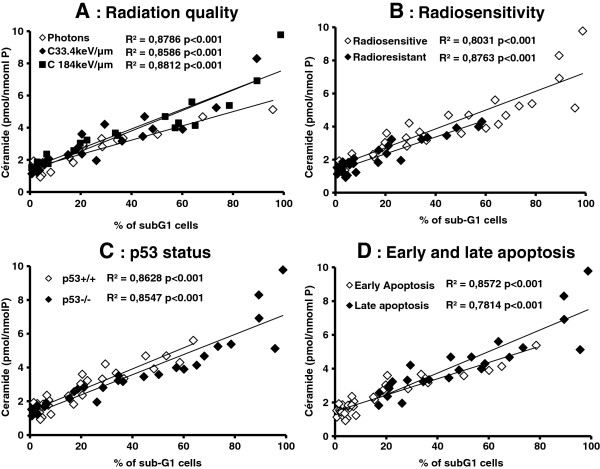
**Pearson correlations between the percentages of cells in sub-G1 after 10Gy of photon or 33.4 or 18 keV/μm carbon ion irradiation as a function of ceramide production.** The comparisons are: **A**: radiation quality; **B**: cell radiosensitivity; **C**: p53-status; **D**: early and late apoptosis.

In order to link ceramide production to the induction of apoptosis, according to the different properties of cells and radiation qualities, a correlation calculation between ceramide concentration and percentages of cells in the sub-G1 phase was made. As shown in Figure 5, highly significant correlations (*p* < 0.001 for all correlations) were obtained regardless of the way of expressing the results. As an example if the results are expressed as a function of p53-status, R^2^ = 0.86 for p53^+/+^ cell lines and R^2^ = 0.85 for p53^−/−^ cell lines were obtained, respectively. The correlation between ceramide and apoptosis were also significant: R^2^ = 0.86 for early and R^2^ = 0.78 for late apoptosis.

These results confirm firstly that ceramide production does not depend on p53-status and secondly that ceramide production is strongly related to apoptosis.

To further determine whether ceramide is released upstream of the commitment phase of apoptosis or if its release is a consequence of membranous alterations in the final apoptosis phase, all cell lines were incubated with Z-VAD-fmk, a total caspases inhibitor. Inhibition of caspases markedly decreased for both early and late apoptosis in all cell lines, regardless of the type of beam applied (Table 2). For example, in radiosensitive SCC61 cells, the ratio of apoptotic cells between treated and control cells felt from 6.9 to 1.7 after carbon ions exposure. Moreover the ceramide concentration was similar in the four cell lines after caspases inhibition whatever the types of radiation. These data indicate that caspases inhibition does not influence ceramide production under our experiment conditions.

**Table 2:** Ratio 10Gy/0Gy, 10Gy + Z-VAD/0Gy + Z-VAD of the percentage of cells in the sub-G1 phase or ceramide production 240 h after photon or carbon ion irradiation

		**X-rays**		**Carbon**	
**Cell lines**	**Ratio**	**Sub-G1**	**Ceramide**	**Sub-G1**	**Ceramide**
SCC61	10Gy/0Gy	6.1	2.6	6.9	3.4
	10Gy + Z-VAD/0Gy + Z-VAD	1.8	2.4	1.7	3.2
SQ20B	10Gy/0Gy	5	1.6	6.3	2.9
	10Gy + Z-VAD/0Gy + Z-VAD	1.9	2.2	1.6	2.6
SF767	10Gy/0Gy	3.9	2.1	6.8	3.4
	10Gy + Z-VAD/0Gy + Z-VAD	1.4	2.7	1.5	3.3
U87MG	10Gy/0Gy	2.8	1.9	2.1	2.3
	10Gy + Z-VAD/0Gy + Z-VAD	1.5	1.7	1.1	2.1

**Figure 6:**
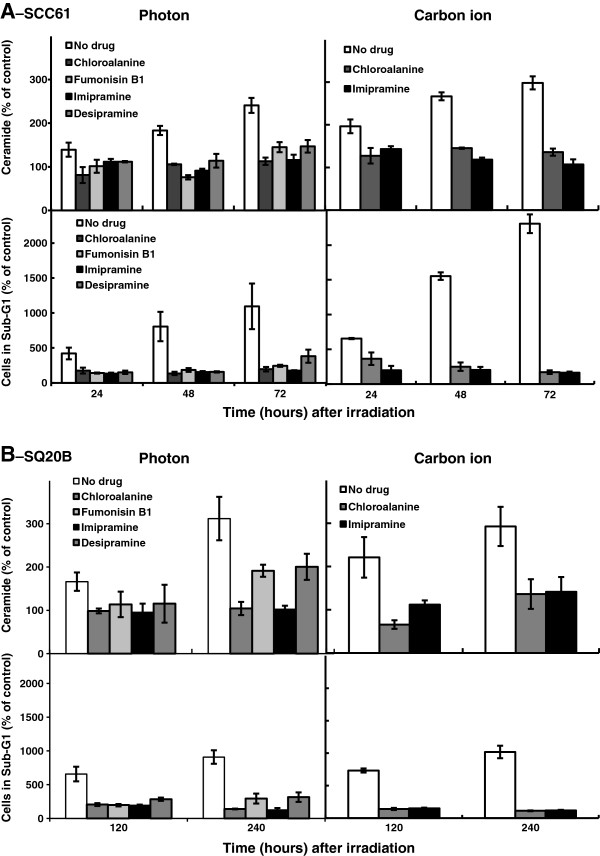
**Kinetics of ceramide production and apoptosis induction in SCC61 (A) and SQ20B (B) cells, with or without pretreatment with 1 mM chloroalanine, 10 μM fumonisin B1, 50 μM imipramine or 20 μM desipramine before 10Gy of photon or carbon ion irradiation.** Values represent the mean ± SD of two independent experiments performed in duplicate.

We next inhibited the two main intracellular pathways leading to ceramide generation in order to determine its impact on the triggering of early (SCC61 cells) and late (SQ20B cells) apoptosis (Figure 6). Chloroalanine, an inhibitor of serine-palmitoyltransferase, and Fumonisin B1, an inhibitor of ceramide synthase were used to inhibit the *de novo* synthesis whereas two inhibitors of acid sphingomyelinase (imipramine and desipramine) were used to inhibit the production of membranous ceramide. As depicted in Figure 6A, pretreatment of SCC61 cells with each drug markedly inhibited ceramide production following high- or low-LET radiation. Consequently, early apoptosis did not occur. For example, 48 h after carbon irradiation, the percentage of apoptotic cells decreased by about 6.2 or 7.8 times after treatment with chloroalanine or imipramine, respectively. In SQ20B cells (Figure 6B), the same treatment inhibited late ceramide production and apoptosis following both types of radiation. When treated, the percentage of SQ20B apoptotic cells (% of control) decreased by approximately 9 times 240 h following carbon ion irradiation compared with untreated cells.

These results demonstrate for the first time that ceramide is responsible for the initiation of early and late apoptosis in response to high-LET irradiation and thus acts as an upstream caspase activator regardless of the p53-status of the cells.

## Discussion

Clinical studies performed on HNSCC or glioblastoma have provided evidence that carbon hadrontherapy is more effective than conventional radiotherapy. However, even if the local control and the survival rates for particles therapy are higher, compared to conventional radiotherapy relapse could always occur. The mechanisms leading to radioresistance are still largely unknown and the need to understand the exact mechanisms involved in the cell death response of different tumor types is essential to improve the treatment.

To date, the p53-mediated apoptosis is the most often occuring pathway responsible for low-LET radiation-induced apoptosis [17], but accumulating data over the past decade have shown that ceramide is the mediator of an alternative apoptotic pathway [4,18]. Ceramide can mediate early apoptosis following low-LET irradiation in different radiosensitive tumor cells [19,20] and is involved in the triggering of apoptosis upstream from mitochondrial dysfunction and caspase activation [4,19]. Intracellular ceramide production is a complex mechanism and may result from either *de novo* synthesis or sphingomyelin hydrolysis by acidic or neutral sphingomyelinases [19,21]. We previously reported that low-LET-induced ceramide production is a complex multiwave event involving both sphingomyelinases and *de novo* synthesis [21]. The new results presented here show in radiosensitive cells the involvement of ceramide in high-LET-induced early apoptosis independently of p53-status. We clearly demonstrated that the production of ceramide occurs within minutes following irradiation. These conclusions are in accordance with previous results published by our group [5] which demonstrated that photon irradiation of the radiosensitive SCC61 cell line results in the triggering of raft coalescence to larger membrane platforms associated with ceramide release. At the opposite in the radioresistant SQ20B cells the lack of early ceramide production result in resistance to early apoptosis following ionizing radiation. Moreover, at the same physical dose, the levels of ceramide production increased proportionally with LET (Figure 1). We thus propose that ceramide would be the mediator of the p53-independent early apoptotic pathway described by Mori *et al.*[13] in response to high-LET radiation. By either inhibiting caspase or ceramide production, we also found that the effect of ceramide occurs upstream of mitochondrial collapse and caspase activation in response to carbon ion irradiation, confirming its major role in the triggering of apoptosis. Our data indirectly confirm that ceramide is produced in several waves: an initial wave involving membranous sphingomyelin hydrolysis and a later *de novo* synthesis that generates the major pool responsible for apoptosis [21].

Mitotic catastrophe has been described extensively as a cell death mechanism during apoptosis in cells with mutation/inactivation of p53 following photon irradiation [22,23]. Cell death results from the premature entry into mitosis because of a compromised G2/M checkpoint. However, Vitale *et al.*[7] recently redefined mitotic catastrophe as a mechanism that senses mitotic failure and responds to it by driving the cell to an irreversible fate, which can be apoptosis, necrosis, or senescence. Consistent with this concept, delayed apoptosis has been shown to occur in the final phase of mitotic catastrophe following low- or high-LET irradiation [11,23]. However, no previous report has considered the role of ceramide in this process. Only Miñano *et al.*[24] demonstrated that the addition of exogenous C2-ceramide, a non-physiological short-chain ceramide, can induce caspase-2 cleavage, which is responsible for the triggering of late apoptosis. The new finding presented in this paper is the existence of a significant correlation between the increase in the amount of late ceramide in radioresistant SQ20B and U87MG cells, and the induction of delayed apoptosis after high and low LET-radiation. Moreover this relationship seems to be independent of p53-status. We therefore suggest that ceramide provides the molecular bridge between mitotic catastrophe and the commitment phase of delayed apoptosis in response to irradiation. This suggestion is supported by our experiments using pharmacological inhibitors, which showed that ceramide acts upstream of the caspases. The two pathways of ceramide production seem to be involved because inhibitors of *de novo* production or acid sphingomyelinase can both inhibit late apoptosis. Moreover we present evidence for the first time that the last step of mitotic catastrophe resulting in delayed apoptosis is mediated through mitochondria alterations after carbon irradiation.

## Conclusion

Ceramide is now emerging as a key factor in the early and late cellular responses to high- and low-LET exposure. Here, we have demonstrated for the first time that carbon and photon irradiation can activate the same apoptotic pathway. This is fundamental information that should be applicable to new promising strategies for cancer treatment based on specific sphingolipid metabolism targeting [25] either after photon or carbon ion exposure. Among others, we have previously reported that increasing endogenous ceramide levels in radioresistant tumor cells can overcome photon resistance to clonogenic and apoptotic cell death [25,26] or restore p53-dependent apoptosis [27]. Our work clearly demonstrates that the modulation of the ceramide pathway will therefore be of interest in the development of future new adjuvant therapies in association with hadrontherapy to improve the treatment of radioresistant tumors.

## Abbreviations

△ψm: Mitochondrial transmembrane potential; HNSCC: Head and neck squamous carcinoma cell; HPLC: High performance liquid chromatography; LET: Linear energy transfer; SF2: Survival fraction at 2Gy

## Competing interest

Non-financial competing interests.

## Authors’ contributions

GA has made substantial contributions to conception, design, analysis and interpretation of data and has been involved in drafting the manuscript. MM has made substantial contributions to conception and acquisition of data. PB-M has made substantial contributions for acquisition of data. DA has been involved in drafting the manuscript. MB has given final approval of the version to be published. RR has given final approval of the version to be published. GT-S has made substantial contributions to conception, interpretation of data. CF has made substantial contributions to conception and interpretation of data. CR-L has been involved in drafting the manuscript. All authors read and approved the final manuscript.

## Pre-publication history

The pre-publication history for this paper can be accessed here:

http://www.biomedcentral.com/1471-2407/13/151/prepub
